# Eye Movements in Mild Traumatic Brain Injury: Clinical Challenges

**DOI:** 10.16910/jemr.15.2.3

**Published:** 2022-06-14

**Authors:** Matthew A. McDonald, Samantha J. Holdsworth, Helen V. Danesh-Meyer

**Affiliations:** Department of Ophthalmology, University of Auckland, New Zealand; Mātai Medical Research Institute, Gisborne, New Zealand; Department of Anatomy and Medical Imaging, University of Auckland, New Zealand; Eye Institute, Auckland, New Zealand

**Keywords:** concussion, sport-related concussion, mild traumatic brain injury, mTBI diagnosis, mTBI epidemiology, mTBI pathophysiology

## Abstract

Mild traumatic brain injury (mTBI), also known as concussion, is a common injury which affects patients of all demographics. There is a global effort to accurately diagnose and identify patients at highest risk of prolonged symptom burden to facilitate appropriate rehabilitation efforts. Underreporting is common with large numbers not engaging with services, in addition to differences in treatment outcomes according to ethnicity, age, and gender. As patients recover, symptomology evolves which challenges rehabilitative efforts with no clear definition of ‘recovered’. This review describes key areas in mTBI such as diagnostic challenges, epidemiology, prognosis, and pathophysiology which serves as an introduction to “Eye Movements in Mild Traumatic Brain Injury: Ocular Biomarkers.”

## Introduction

Mild traumatic brain injury (mTBI), commonly referred to as concussion, is a complex neurobehavioural phenomenon resulting from mechanical trauma (16). Despite increased knowledge of the biomechanics and pathophysiology of concussion, no standardized biomarkers exist (either clinical or serological). Health professionals currently rely on symptom reporting which many patients are not willing to disclose (e.g. athletes, military personnel, or patients pressured into return to work). Likewise, prognosis is variable and it is not possible to predict which patients will require prolonged rehabilitation therapy. In recent years, researchers have shifted their focus to eye tracking due to the widespread neural pathways responsible for ocular motor control. These ocular motor abnormalities may serve as a useful tool in everyday clinical practice for not only diagnosis, but also serve as a biomarker for recovery (94). This review will provide a comprehensive introduction to “Eye Movements in Mild Traumatic Brain Injury: Ocular Biomarkers”, to summarize the current state of mTBI epidemiology, diagnosis (including subtypes), prognosis, and pathophysiology.

### mTBI Diagnosis and Challenges

A single unifying diagnostic nosology for classifying mTBI represents one of the greatest challenges within the field. The term concussion and mTBI are often used interchangeably, however, some authorities suggest that concussion should be considered a subset (milder form) of mTBI, although there is no consensus of such a classification (66). Currently there are no distinct symptom diagnostic criteria that differentiate concussion from mTBI. Current guidelines from the Centers for Disease Control (CDC) promote the single term “mild traumatic brain injury”, instead of concussion (61; 84).

Although there is extensive discussion regarding the operational definition of mTBI, the definition by the American Congress of Rehabilitation Medicine, revised by the World Health Organization (WHO), seems to be increasingly accepted by clinicians and researchers in the field (56). The definition of mTBI requires a Glasgow Coma Scale score between 13 and 15 at 30 minutes post-injury, and one or more of the following symptoms: <30 min loss of consciousness; <24 hours post-traumatic amnesia; impaired mental state at time of accident (confusion, disorientation, etc.); and/or transient neurological deficits (68). Neuroimaging is typically normal as standard techniques are not sensitive enough to detect damage in the majority of cases (10% sensitivity for CT and 30% for MRI) (14; 71; 81).

Sport-related concussion (SRC) is considered by some investigators to have its own nosological framework. The Consensus Statement on Concussion in Sport at the 4^th^ International Conference on Concussion qualifies SRC as a direct blow to the head, face, neck, or elsewhere on the body with an ‘impulsive’ force transmitted to the head (68). Typically, this results in rapid onset of short-lived impairment of neurological function that resolves spontaneously. However, in some cases, signs and symptoms evolve over a number of minutes to hours. Standard neuroimaging is normal as the acute clinical signs and symptoms are considered to largely reflect a functional disturbance rather than a structural injury.  It is important to note that clinical features should not be explained by drug, alcohol, medication, other injuries (e.g. cervical injuries or peripheral vestibular dysfunction), or other comorbidities (e.g. psychological factors or coexisting medical conditions). Loss of consciousness is not a requirement. While resolution of the clinical and cognitive features typically follows a sequential course, some cases experience a prolonged symptom burden.

Hence, one of the challenges of mTBI research is that the present definition encompasses a broad spectrum of injury. From the above definitions it is clear that the limits of the definitions may overlap with ‘moderate’ TBI at the upper end and trivial head trauma at the lower end. This has a significant impact in interpreting the research in particular with respect to prognosis and management.

## Epidemiology

**Figure 1.**
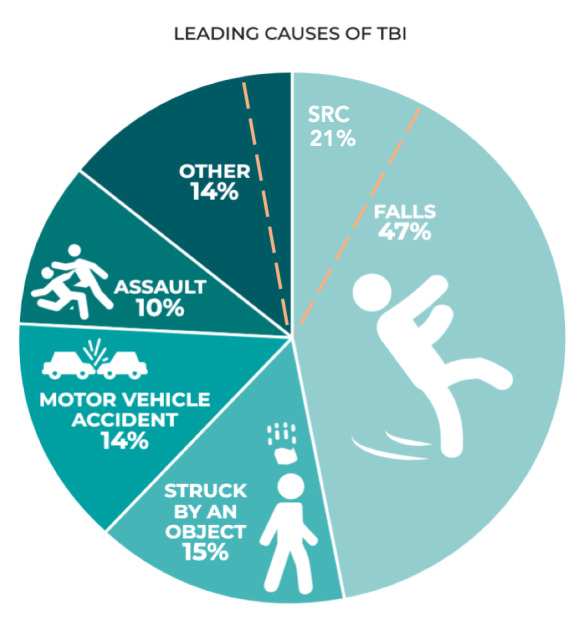
Leading causes of TBI in the 2014 CDC TBI surveillance report (published in 2019) based on hospital admissions in the US (74). Sport-related concussion (SRC) has been added by authors (dashed orange line) based on community data from Theadom et al. (90).

Over 50 million people suffer traumatic brain injury each year (at least 6 per 1,000 globally) (39; 62) and it is estimated that half the global population will experience a form of TBI during their lifespan (62). Mild traumatic brain injury forms 60-95% of TBIs (62) when averaged across the US, Middle East, Eastern Europe, and Asia with a global incidence of 939/100,000 for all-cause TBI when averaged across African region, Latin America, US/ Canada, Eastern Mediterranean, Europe, Southeast Asia, and Western Pacific (23). In higher income countries, elderly fall-related TBIs are increasing, whilst trauma from road traffic accidents is increasing in lower-income countries (Figure 1) (62). UK data suggests 30% of people aged over 65 will fall at least once per year and for those over 80, the risk is over 50% (25). Likewise, the Center for Disease Control in the USA cites a prevalence of 27.5% of people aged over 65 per year (35.6 million falls per year) (72). Although specific data is not provided with regard to injury subtype following these events, a range of TBI will inevitably arise due to direct or indirect transmission of force.

**Figure 2.**
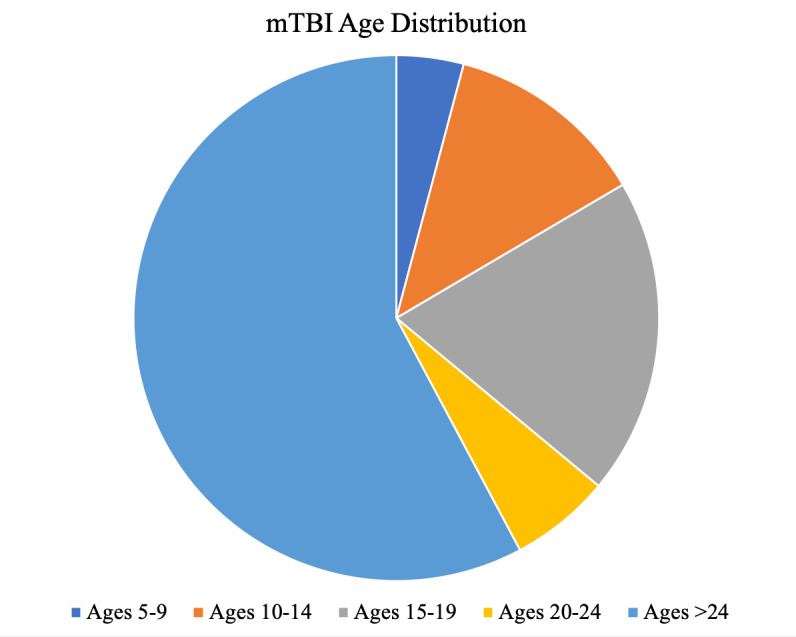
Distribution of mTBI incidence. Ages 5-24, less than two decades, account for 42% of the entire mTBI population, with ages 15-19 most dominant. Data adapted from (99).

A meta-analysis of 15 prevalence studies (25,134 adults) found that 12% of the sample experienced TBI with loss of consciousness, with men being at more than double the risk of women (29). Another epidemiological review of US health insurance companies by Zhang and colleagues analyzed 8,828,248 patients and reported an overall incidence of TBI of 4.9%, with a third falling between ages of 10-19 (29% overall rate of loss of conciousness) (99). These numbers increased by 60% from 2007 to 2014 when the study was conducted, reflecting both population growth and increased health-seeking behaviour (99). Of these subjects, male gender comprised 55% of the sample with the highest incidence of mTBI between the ages of 15 to 19 (16.5/1000), followed by ages 10-14 (10.5/1000), ages 20-24 (5.2/1000), and 5-9 (3.5/1000) (Figure 2). This sample consisted of 56% being diagnosed in the emergency department and 29% at primary care (the remainder were in urgent care and inpatient settings) (99). A major limitation to this review was its inability to detect milder brain injuries in other community settings, such as sports fields, where these are under-reported and hence remain undetected.

A significant burden of mTBI is related to sports-related concussion (68). In the United states, this incidence is at least 15.4/100,000 people per year based on an epidemiological study of sport-related mTBI presenting to hospital departments (83). The majority of studies with this type of selection bias from hospital data cite sport-related concussion as anywhere from 5.4% (Austria) (65) to 26.7% (USA) (57) of all-cause mTBI. These figures are conservative as they do not account for presentations to other health services and unreported mTBI.

A community-based study exploring multiple sources of referrals revealed a significantly higher incidence of 170/100,000 and sport-related causes comprised 21% of these (90). In a cohort of athletes, a third experienced previous undiagnosed mTBI (69) and it is estimated as many as 70% of head injuries go unreported (86). Similarly, in a survey of 133 rugby union players (age under 20), 48% reported experiencing at least one mTBI (mean 2.25) for which half did not seek medical attention (6). This is particularly relevant in adolescent populations; those who began contact sports before the age of 12 were deemed twice as likely to have long term dysregulation in behaviour and three times as likely to experience apathy or depression (2).

### Gender

mTBI predominantly impacts young males (66% to 76% of all cases) which is due to higher rates of contact-sports and riskier maneuvers such as tackling (88). While male athletes have a higher concussion incidence overall, concussion incidence for gender-comparable sports is higher among females (20; 24; 31; 58; 63). Further studies on gender differences in mTBI have shown females report a higher number of mild symptoms at baseline testing (21). Colvin and colleages noted slower reaction times, increased symptoms, and lower neurocognitive scores in a cohort of 234 soccer players, aged 8 to 24 years (141 females, 93 males) (18) while another study involving 260 youth (adult cohort: 47 males, 31 females, aged 18-59; paediatric cohort: 97 males, 40 females, aged 4-17) concluded prolonged symptoms were more frequent in adult females, but not minors (73). These limited gender studies highlight the need for more research in this area.

### Ethnicity

Some studies have investigated the relationship between racial disparities and mTBI as well as sport-related concussion and have identified that there appears to be differences in concussion incidence, awareness, outcome, and morbidity. African American children under the age of 4 years old who sustained TBI have been shown to have mortality rates twice those of Caucasians (53). Other research has shown African American athletes are at a greater risk of neurocognitive impairment (over twice as likely to experience at least one cognitive decline measure on the ImPACT test along with lower processing speed compared to their baseline) 7 days following sports-related concussion (50).

Inequities in healthcare based on ethnicity have been identified in the entire spectrum of mTBI. African American patients were less likely than Caucasian patients to have emergency department visits for head injuries and were less likely to be diagnosed with a concussion during an emergency department visit (31). African American children also have higher rates of concussion/mTBI from assault compared to sports injuries. Bloodgood et al. have also highlighted that African Americans and Hispanics in the USA had less awareness of mTBI than non-Hispanic Caucasians which may lead to underreporting (12) in addition to differences in cognitive-related scores between African Americans and Caucasian Americans (95). This may further bias population-based studies from higher under-reporting in these groups. The situation is echoed in New Zealand where underreporting is highest amongst the Māori population who were also found to have a 23% greater risk of mild TBI than New Zealand Europeans (27). Of those that are reported, they are more severe in nature and have lasting effects, including increased health care needs (49).

Future epidemiological studies are required to investigate incidence longitudinally with consideration of factors such as age, gender, ethnicity, and particular risk-factors in sport-related concussion such as level of competition (elite vs non-elite) and player position which is known to affect risk (30).

## mTBI Subtypes

mTBI is a heterogenous injury resulting in diverse clinical presentations which cluster into broad domains (68). These domains are also dynamic; patients who present with one dominate symptom cluster may evolve into another as recovery progresses. The five most commonly recognized subtypes are described below from a 2019 literature review and meta-analysis by experts from the 2015 “Targeted Evaluation and Active Management” meeting (60):

**Table 1:** Subtype as described in (60).

Subtype	Symptoms/ Risk Factors	Prevalence
Cognitive	Impairment in attention, reaction time, memory (storage, retrieval, and working), processing speed, thought organization, and behaviour.	32% in a paediatric cohort and 40% in adults
Headache/ Migraine	Patients with a history of chronic headaches or migraine were considered high risk of exacerbation following mTBI	52% in a paediatric cohort and 38% in adults.
Vestibular	Impairment of movement, proprioception, and balance, resulting in dizziness, ‘fogginess’, nausea, vertigo, and ‘lightheadedness’. A vestibulo-ocular subtype consists of abnormal vestibulo-ocular reflex, visual motion sensitivity, gait impairment, and balance issues.	50% in a paediatric cohort and 25% in adults.
Anxiety/ Mood	Nervousness, experienced heightened emotions, ruminating, feeling overwhelmed, depression, hopelessness, fatigue, and anger/ irritability.	30% in a paediatric cohort and 23% in adults.
Ocular motor	Asthenopia (‘eye strain’), ‘tired eyes’, difficulty with near or distance (from impaired vergence and accommodation), photophobia (sensitivity to light), frontal headaches, blurred vision, pressure around the eyes, and vision-related nausea. These issues arise from difficulties in obtaining and processing visual stimuli from impaired eye movements (saccades and smooth pursuits; see section below).	34% in a paediatric cohort and 34% in adults (although this showed a large range).

Concussion-associated conditions are common symptoms which may be present irrespective of subtype. These include sleep disturbance and cervical strain. These patients may experience insomnia or hypersomnolence (60). Cervical strain consists of neck pain, stiffness, weakness, or chronic occipital/ suboccipital pain, which is often caused by whiplash injury (13), particularly prevalent in wheelchair athletes (37).

However, these domains (and assocated conditions) have recently been challenged following a systematic review and meta-cluster analysis by Langdon and colleagues of 5592 athletes across 22 carefully selected studies (52). Their analysis revised these five subtypes (based on symptomology from the Sport Concussion Assessment Tool, 5th edition, SCAT-5 (36) which correlated to clinical outcomes. Their rationale was clear: to provide a unified evidence base toward individualized mTBI management and treatment to enhance recovery and reduce prolonged symptom burden.

**Table 2:** Subtypes as described in (52)

Subtype/ “Cluster”	Symptom	Association	Prevalence
Migraine	Headache, sensitivity to light/ noise, and nausea	Concomitant cognitive, balance, and vestibulo-ocular motor symptoms which correlated to prolonged recovery	24%
Cognitive-emotional	Difficulty concentrating, remembering, ‘fogginess’, increased emotion, irritability, sadness, nervousness, and ‘feeling slowed down’.	Prolonged recovery, balance deficits, and greater total symptom severity scores	19%
Sleep-emotional	Trouble falling asleep, sleeping less (and more), increased emotion, irritiability, sadness, nervousness	Prolonged recovery, lower sleep quantity, cognitive impairment, balance impairment, and greater total symptom severity scores.	21%
Neurological	Blurred vision, vomiting, neck pain, pressure in the head, visual problems, and double vision.	Associated with vestibulo-ocular motor screening symptoms.	2%
Undefined feelings cluster	“Not feeling right” and confusion	No evidence to correlate this to a specific clinical outcome.	Not quantifiable in literature

## Prognosis and Risk Factors

Recovery from mTBI is variable depending on the population studied and the definition of mTBI. The two week recovery of patients with sport-related concussion has been reported to range from 50% to 90% (40). At two months, 4-12% of young adults will still be symptomatic (9; 45). Long-term outcomes are equally variable. In the primary care setting, half of patients experienced four or more post-mTBI symptoms at 12 months with 10.9% of participants suffering from low cognitive functioning and increased levels of mood disturbance (anxiety, depression) (89). In this group, risk factors for prolonged recovery were a previous history of brain injury, living alone, ethnicity (non-caucasion), alcohol and medication use, and female gender. Even after adjusting for gender-related differences, evidence suggests an ongoing symptom burden in females of almost twice as long as males (8), attributable to reduced neck-head segment mass (91), differences in cerebral blood flow (26), and reponse to injury on neurobehavioural assessments (15; 21; 32). Other groups have also correlated severity of symptoms post-injury, repeat injury, subacute development of headaches and mood disturbances, age (children and adolescents), and pre-existing mental illness to prolonged symptom burden (8; 42; 45; 85).

In a cohort of 91 students, aged 13-19, a third of those who reported problems returning back to class following an mTBI had a history of previous head trauma. Similarly, a third of students struggled with return to school (57% of students took over 10 days recover and 29% took over 21 days to recover). Of these, vision problems were correlated to a 2.5x risk of difficulty at school (7). Likewise, a cohort of 247 patients aged 5-18 revealed a time to return-to-school of 12 days, but without accommodations (e.g. reduced hours, extra help) this was 35 days (19). Adolescents were not symptom free until 64 days and were cleared for sport at 75 days. At 4 weeks post-injury, 73% of these patients were symptomatic and 61% showed a decline in grades that year.

There is a paucity of literature in the elderly population’s prognosis following mTBI, but studies in this area suggest poor outcomes on cognition and independence following an injury (78). A prospective study spanning two years post-injury found early cognitive decline more prevalent in the elderly population following head injury (mean age 69.9 +/- 11.5) even when controlling for pre-morbid cognitive status (35). Another study showed adults over 65 years at one year post injury had high rates of low mini-mental state examination score (62% scored < 24) with 32.6% reporting mild to moderate disability following their injury and 56.8% meeting the criteria for ‘postconcussion syndrome’ (22).

### Postconcussion Syndrome (PCS)

This controversial clinical entity has been defined as a constellation of physical, cognitive, behavioral, and emotional symptoms that persist beyond 3 months post-injury. Symptoms include headache, fatigue, visual changes, balance issues, confusion, dizziness, insomnia, neuropsychiatric symptoms, and concentration deficits (10). Both the International Classification of Diseases, 10th revision (ICD-10), and the DSM-IV (which refers to PCS as ‘postconcussional disorder’) have different clinical criteria (3; 97). When utilized on the same patient cohort, the incidence has been shown to vary depending on which criteria was used, despite no difference in outcome measures of psychiatric symptoms, quality of life, and community engagement (67). It is unclear whether this is related to ongoing trauma-related neuropathological changes, a secondary phenomenon such as premorbid conditions (e.g. migraine, mental illness), or perhaps both (54).

The pathophysiology of PCS remains poorly understood. Some theories suggest it has a psychogenic origin (1; 28; 41; 43; 55; 59), while others suggest that it is related to persistent microstructural (46; 82), autonomic (17; 51; 70) and metabolic alterations (33) in the brain.

This evidence is supported by studies correlating diffusion MRI abnormalities (a surrogate for white matter tract damage) to ocular motor dysfunction in patients with ongoing symptom burden. Maruta and colleagues suggested that disrupted white matter integrity in the right anterior corona radiata, uncinate fasciculus, and genu of the corpus callosum correlated to decreased gaze accuracy in smooth pursuit eye tracking paradigms (64). Taghdiri’s group found that diffusion measures of the left uncinate fasciculus mediated the relationship between time of last concussion to number of self-paced saccades (decreased numbers in postconcussion cohort) with a further correlation between the left cingulum to total symptom burden and number of self-paced saccades (87). Tyler and colleagues’ small cohort of 12 mTBI patients (2 months to 35 years post-injury) suggested that their increased saccade latency, slower velocities, and reduced convergence and divergence velocities (compared to 11 age-matched controls) were due to a 50% reduction in functional MRI signal (blood-oxygen-level-dependent-contrast) in brainstem nuclei responsible for eye movements (92). There is clearly emerging evidence in this area to suggest ongoing pathophysiological change in these patients, but more studies are required with larger cohorts, more robust inclusion criteria (e.g. mTBI criteria, time since injury, recruitment), and standardized assessments of eye movement dysfunction in both methodology and analysis.

Arguments for premorbid mental illness or maladaptive psychological phenomena following mTBI cite significant overlap and similarities to somatization observed in psychiatric disorders such as depression, anxiety, and post-traumatic stress disorder (PTSD). The DSM-V classification for major depressive disorder includes difficulty concentrating, headaches, indecisiveness, loss of energy, anxiety, and insomina, all of which are mTBI symptoms (4). A study evaluating postconcussive symptoms in patients with depression showed that 78% experienced poor concentration, 86% fatigue, 59% headaches, 41% nausea, 66% nervous/ tense feelings, and 78% with disordered sleep (41). In this group, 90% of patients with depression met self-reported criteria for postconcussion syndrome (41). Symptom assessment alone may therefore prove ineffective in sufficiently classifying these patients. Additionally, the label of “postconcussion syndrome” may either validate a patients’ sick role to prevent further recovery attempts or provide false reassurance that their symptoms may spontaneously resolve, preventing further investigation or treatment (84). “Ongoing (or prolonged) symptom burden” may serve as a more useful descriptor.

Overall, PCS remains a contentious diagnosis among health professionals despite an increase in literature rationalizing persistent neurological dysfunction. Individuals must continue to be treated on a case-by-case basis, accounting for multiple health-related factors.

## Pathophysiology

Following an mTBI, a series of complex biochemical, metabolic, and microstructural changes occur. Mechanical trauma (from either acceleration/deceleration, blunt trauma, or rotational forces) causes shear and stress forces within the brain parenchyma which disrupts cellular membranes. This leads to extracellular shifts of potassium, intracellular shifts of sodium and calcium, glutamate release, and diffuse neuronal depolarization (34; 47; 75). Global depression in neuronal function ensues which is attributed to hyperacute mTBI symptomology (e.g. loss of consciousness, amnesia, confusion, drowsiness). An ‘energy crisis’ follows where cellular membrane ion pumps use more adenosine triphosphate than is available to restore homeostasis leading to hyperglycolysis. Excessive free radical production from altered metabolism leads to a well-recognized window of increased cerebral vulnerability where insufficient recovery time places the patient at risk of exponentially more serious damage from a second mTBI (76; 77; 93).

After the insult, disruption to the blood-brain barrier results from cerebral microvasculature trauma and loss of junctional adhesion proteins (98), causing a downstream cascade of inflammation from blood-borne factors, initiating microglial activation and pro-inflammatory cytokines (38; 44; 47). The proinflammatory response is accelerated by glutamate release as the immune system mounts a response to oxidative stress (referred to as immune-excitotoxicity) (11).

Microstructural changes to cytoskeletal architecture of dendrites, astrocytes, and axons (in particular microtubules and neurofilaments) leads to impaired neurotransmission. In particular, unmyelinated axons are at greater risk of damage which explains why younger brains may be at greater risk of long term sequelae (79). In addition, repeat trauma also prevents synaptic plasticity (reorganization of axons) and neuronal recovery (5; 96). The degree of axonal disruption, as dictated by the severity of injury, positively correlates to the degree of neurobehavioural disruption (i.e. symptomology) which, in turn, leads to the degree of network disruption (34; 48). As recovery occurs, long fiber tracts and hubs (intersections of functional networks) continuously rearrange themselves which manifest as evolving symptomology (48).

For an in-depth review of this topic, refer to Romeu-Mejia et al. and Giza et al. (34; 80).

## Conclusion

mTBI is a common injury which represents an unmet health need on a global scale. There are challenges in not only identifying and subsequently diagnosing these patients, but also grouping into sub-types for appropriate referral pathways. Further clarification of definitions and criteria in mTBI is required for unified efforts (both clinically and for researchers) in this area. In future, ocular motor characteristics may serve as a diagnostic aid given emerging evidence which positively correlates to white matter tract disruption in the brain. This is particularly relevant to a large group of mTBI patients suffering from visual symptoms. Measuring the scale of the epidemiological burden of mTBI in communities is difficult with large variation from hospital-based and primary care (general practice) surveys. This is compounded by underreporting in sports leagues and adolescent populations. Racial disparities in health literacy, health-seeking behaviours, and treatment outcomes reflect wider societal issues. Further studies exploring these issues in greater detail may elucidate contributing factors to facilitate policy and culture change. In addition, the lack of literature on mTBI in the elderly is alarming, considering the prevalence of falls in this population. Where symptoms may be difficult to evaluate in the cognitively impaired, biomarkers such as quantitative eye tracking may prove useful. When studying mTBI trajectories across populations, prognosis is varied as diagnosing a patient as ‘recovered’ results in discharge from services and may not reflect complete resolution of symptomology or physiological recovery. This is evident from primary care surveys at one year and outcomes in schools as mentioned above. The spectrum of ongoing symptom burden and label of postconcussion syndrome may not be helpful with notable overlaps in symptoms of mental illness. Unravelling the pathophysiology and identifying a biomarker in neural recovery is key to improved service provision. Overall, mTBI is a common injury with significant gaps in both our primary understanding of this condition and treatment modalities.

### Ethics and Conflict of Interest

The author(s) declare(s) that the contents of the article are in agreement with the ethics described in http://biblio.unibe.ch/portale/elibrary/BOP/jemr/ethics.html and that there is no conflict of interest regarding the publication of this paper.

### Acknowledgements

This research was supported by the Health Research Council of New Zealand through the Clinical Research Training Fellowship.
